# Poisons as Required

**Published:** 1891-01-10

**Authors:** 


### THE DOCTOR'S ARMCHAIR.

"POISONS AS REQUIRED."

MR. PUNCH relates his experiences among the Planets in his Christmas number. Just as Mr. Labouchere extracted the truth from all and sundry by means of hypnotism, so Mr. Punch had on his finger a truth-compelling ring during his planetary experiences. However great a rogue he might meet, he had only to give his ring a turn on the finger, and, lo! the rogue turned honest; not only honest, but garrulous, too; and straightway Mr. Punch became the possessor of all the truth he wanted. There was a certain wine merchant in Mercury who very politely offered the visitor a taste of his wares, with a view to business. Beautiful to look at, thought Mr. Punch, as he held the glass up to the light; but, with a wisdom born of much contact with earthly affairs, he wondered whether the gold was as fine as the glitter. Like a skilful lawyer, he gave his ring a turn, and then asked a leading question of his host. "How do you manage it?" said he. "How do I manage it?" replied the man of wine, compelled to truthfulness by the power of the ring. "Why, my dear sir, there's not much difficulty about that. I just make it myself. Listen to my receipt:—

> "Potato spirit—that the "body" finds;
> And then, as for colour,
> Be it brighter or duller,
> You see I am supplied with several kinds;
> And as to flavour, I get that desired,
> By adding various poisons as required."

Science has a nose for honesty as well as for knavery. A man who really loves his country and his kind feels a little inclined to be angry when he hears or reads a universal denunciation of his fellow-men. Satire has its merits; and from the earliest times it has been a favourite instrument in the hands of the more cynical moralist. It has not, therefore, the merit of originality. It is cheap, if that be a recommendation; and it is a sort of instrument which can be used without much previous training. Of course, if the satirist is to excel in his art, he must have both natural aptitude and acquired skill. But the question is, Is it worth while for a really capable man to devote himself to the acquisition of the satirical art? Science, which has earned some right to speak, says it is not; at any rate, nobody ever becomes scientific by the use of the methods of satire. Carlyle, who spoke from long experience and practice, finally declared his conviction that satire was of the devil. Probably it is; at least, in those cases where an individual is not only a satirist, but a satirist only.

Mr. Punch imagined himself a man with a ring. The present writer has often tried to turn himself into a dog with a nose. No healthy dog has a dishonest nose. He does not smell vile odours where there are none. A medical practitioner necessarily sees many varieties of wines, and, of course, of teas, coffees, cocoas, beef juices, infants and invalids' foods, *et hoc genus omne*. He meets also, if he be a practical man, a good many wine merchants, and other persons of the class of purveyors for the material needs of the community. As a young man of some little acquaintance with the Greek and Roman satirists, King Solomon, *Punch*, *Truth*, and the daily newspapers, he probably begins his professional life with the idea that seven businessmen, of the class named, in every ten are cheats, who adulterate their goods; that two are doubtful; and that only one of the whole half-score is honest. Now, how does his actual experience on these points compare with the ideas which were in his mind when he began practice, and which, as we have seen, were so largely gathered from literary and other similar sources? This is a question well worth asking and well worth considering. For why should we look upon a large proportion of our fellow-men as knaves and scoundrels unless we are compelled by facts to do so? What right has a newly-fledged university graduate, or a Grub street philosopher in a rusty frock-coat, to take it for granted that the current slanders against men of business, of which the newspapers are periodically so full, are as true and accurate as scientific research can make them? A dozen men whose character and capital consist of nothing but pens, paper, and a limited supply of ink, can blacken the reputation of a whole generation, and the pitch may not be washed off for centuries.

Speaking from an experience of fifteen or twenty years, one medical man, at any rate, is able to say that he has not found his fellow-men of the business class half so black as they have been painted. Wines, which are so commonly ordered for sick persons, are seldom or never the poisons they are said to be, unless they are purchased at poison prices. The poor, who cannot afford to pay for good wines and spirits, should leave such things entirely alone if they cannot procure them from charitable friends. An old-established wine merchant admitted to the writer quite recently that poisonous wines and spirits are undoubtedly manufactured; but then they are manufactured because there is a demand for them on the part of people who cannot afford to pay for *bonâ fide* wines and spirits. Those persons who can pay for genuine articles are just as sure of getting them honest and good as they are of getting honest and capable medical practice when they can offer reasonable fees for it. Exactly the same may be said of teas, coffees, cocoas, beef juices, infants and invalids' foods, and their makers. All these things can be, and are, obtained of the highest order of excellence by people who are able and willing to pay for them according to their market value.

There are two reasons for taking up a question of this kind. The first is that it is important as a matter of everyday business that people should have a just measure of confidence in the food they eat and the medicines they take. Many persons are unceasingly haunted by suspicions that they are eating alum in their white bread, beans instead of wheat in their corn-flour, pease-meal in their cocoa, and chicory in their coffee; that the wines they drink are made of gooseberry and rhubarb, and that the spirits are mainly fusel oil; that even their medicines are so liberally adulterated that the doctor can never rely with confidence upon producing the therapeutic results which he is so anxious to bring about. So far as the prosperous classes are concerned such suspicions are a foolish nightmare, while, as regards the poor, they have the matter very largely in their own hands, in that they can, if they will, do without wines and spirits and other luxuries, for which they cannot afford to give an adequate price.

Our second object in dealing with this question is to raise a protest against the reckless blackening of the character of our fellow-countrymen. Literary persons love effective paragraphs; science and honesty love truth. It is quite as possible to be a knave in a study or a newspaper office as in a factory or a vineyard. He who exaggerates and distorts facts for the sake of producing a larger and more striking effect than the case warrants, may be a very clever person from a literary point of view, but he is certainly a cheat and a knave, and would, if he dealt in mustard or in tea instead of ink and paper, be an adulterator of his wares. He is often, in fact, a much worse man than the cocoa manufacturer who mixes pease-meal with his cocoa, if there be such a person, or the tea merchant who blends cheaper teas with dearer in order to sell at a lower price. For he not only spreads suspicion abroad to the injury of business and the unnecessary worry of the purchaser, but he recklessly destroys the character of his fellow-countrymen in the eyes of other countries and of future generations.

But what surprises the present writer most of all is the want of originality in the Christmas issues of *Punch, Truth,* and other satirical papers. Year after year, and decade after decade, they are ceaselessly harping upon the same string and bringing out the same thin, squeaking, and melancholy notes. Can it be that the writers are so poor in honesty and truthfulness themselves that they cannot believe in the honesty and truthfulness of other people? That we do not think for a moment—we know better. But we ask the question in order that we may pull them up short, and make them consider how they themselves would like to be taken for shams, and cheats, and knaves. Our experience of life is that the vast majority of British people of all classes are sound-hearted; that by preference they do right; that when they do wrong they do it under an inner protest, and with disgust and remorse; and that no class of people in this country are worthier of confidence and respect than the merchant and manufacturing class, who do the greater part of the work of the world and bear most of its burdens. A doctor is necessarily much behind the scenes, and sees a great deal of the inner working of men's and women's hearts; and it is a doctor who dares to make public that he has a much better opinion of human nature, and of British human nature in particular, as the result of personal experience, than he had in his youthful days when he gained his knowledge almost exclusively from books. In the future the scientists, it may be hoped, will kick out the satirists, and thereby make the cup of life much more wholesome drinking.

